# *trans*-Acetyl­dicarbon­yl(η^5^-cyclo­penta­dien­yl)[tris­(furan-2-yl)phosphane-κ*P*]molybdenum(II)

**DOI:** 10.1107/S160053681302059X

**Published:** 2013-07-31

**Authors:** Matthew T. Whited, Julia G. Bakker-Arkema, Julia E. Greenwald, Lucas A. Morrill, Daron E. Janzen

**Affiliations:** a Department of Chemistry, Carleton College, 1 N. College St, Northfield, MN 55057, USA; b Department of Chemistry, St Catherine University, 2004 Randolph Ave., St Paul, MN 55105, USA

## Abstract

The title compound, [Mo(C_5_H_5_)(C_2_H_3_O)(C_12_H_9_O_3_P)(CO)_2_], was prepared by reaction of [Mo(C_5_H_5_)(CO)_3_(CH_3_)] with tris­(furan-2-yl)phosphane. The Mo^II^ atom exhibits a four-legged piano-stool coordination geometry with the acetyl and phosphine ligands *trans* to each other. The O atom of the acetyl ligand points down, away from the Cp ring. In the crystal, mol­ecules form centrosymmetrical dimers *via* π–π inter­actions between furyl rings [the centroid–centroid distance is 3.396 (4) Å]. The dimers are linked by C—H⋯O hydrogen bonds into layers parallel to (100).

## Related literature

For synthetic details for a related complex, see: Gladysz *et al.* (1979 ▶). For related structures, see: Churchill & Fennessey (1968 ▶); Barnett *et al.* (1972 ▶); Michelini-Rodriguez *et al.* (1993 ▶); Adams *et al.* (1997 ▶, 2000 ▶); Whited *et al.* (2012 ▶).
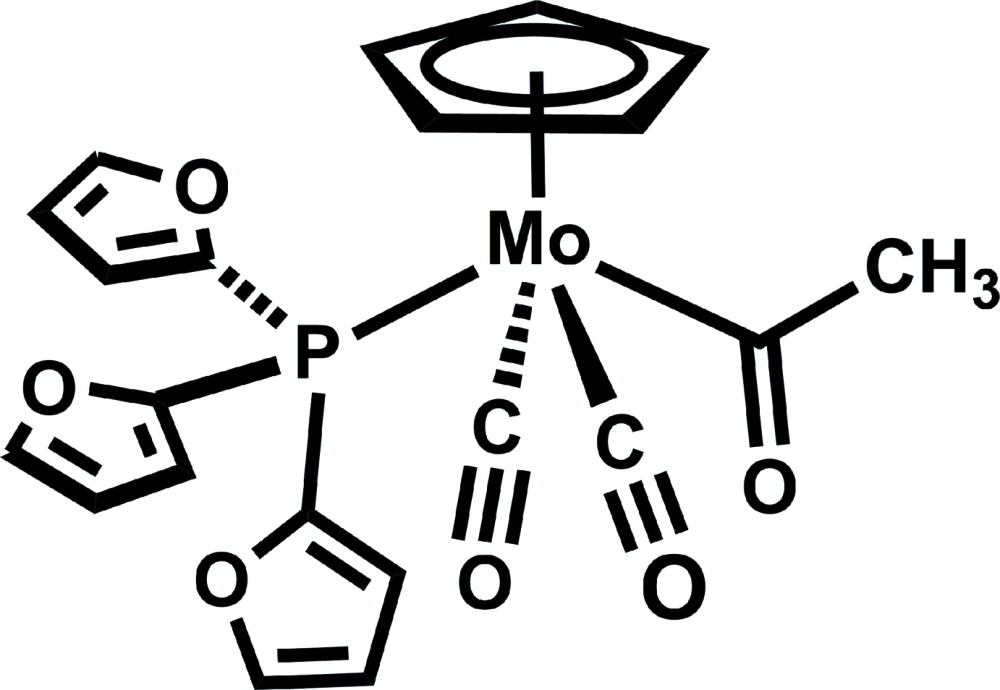


## Experimental

### 

#### Crystal data

[Mo(C_5_H_5_)(C_2_H_3_O)(C_12_H_9_O_3_P)(CO)_2_]
*M* *_r_* = 492.28
Monoclinic, 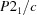

*a* = 8.050 (2) Å
*b* = 15.762 (4) Å
*c* = 16.073 (4) Å
β = 102.852 (8)°
*V* = 1988.4 (9) Å^3^
*Z* = 4
Mo *K*α radiation
μ = 0.78 mm^−1^
*T* = 173 K
0.24 × 0.17 × 0.15 mm

#### Data collection

Rigaku XtaLAB mini diffractometer
Absorption correction: multi-scan (*REQAB*; Rigaku, 1998 ▶) *T*_min_ = 0.709, *T*_max_ = 0.890
16222 measured reflections
4539 independent reflections
3799 reflections with *I* > 2σ(*I*)
*R*_int_ = 0.044

#### Refinement

*R*[*F* ^2^ > 2σ(*F* ^2^)] = 0.035
*wR*(*F* ^2^) = 0.075
*S* = 1.06
4539 reflections
263 parameters
H-atom parameters constrained
Δρ_max_ = 0.55 e Å^−3^
Δρ_min_ = −0.52 e Å^−3^

### 

Data collection: *CrystalClear* (Rigaku Americas and Rigaku, 2011 ▶); cell refinement: *CrystalClear*; data reduction: *CrystalClear*; program(s) used to solve structure: *SIR2004* (Burla *et al.*, 2005 ▶); program(s) used to refine structure: *SHELXL97* (Sheldrick, 2008 ▶); molecular graphics: *CrystalStructure* (Rigaku Americas and Rigaku, 2010 ▶); software used to prepare material for publication: *CrystalStructure*.

## Supplementary Material

Crystal structure: contains datablock(s) General, I. DOI: 10.1107/S160053681302059X/kq2007sup1.cif

Structure factors: contains datablock(s) I. DOI: 10.1107/S160053681302059X/kq2007Isup2.hkl

Supplementary material file. DOI: 10.1107/S160053681302059X/kq2007Isup3.cdx

Additional supplementary materials:  crystallographic information; 3D view; checkCIF report

## Figures and Tables

**Table 1:** Selected bond lengths (Å)

Mo1—P1	2.4189 (8)
Mo1—C1	2.253 (4)
Mo1—C3	1.968 (3)
Mo1—C4	1.982 (3)
Mo1—C5	2.341 (4)
Mo1—C6	2.332 (4)
Mo1—C7	2.359 (3)
Mo1—C8	2.374 (4)
Mo1—C9	2.382 (4)
O1—C1	1.227 (4)
O2—C3	1.150 (4)
O3—C4	1.148 (4)

**Table 2:** Hydrogen-bond geometry (Å, °)

*D*—H⋯*A*	*D*—H	H⋯*A*	*D*⋯*A*	*D*—H⋯*A*
C8—H8⋯O1^i^	1.00	2.38	3.324 (4)	158
C11—H11⋯O1^i^	0.95	2.40	3.166 (5)	137

Symmetry code: (i) 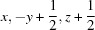.

**Fig. 1.**
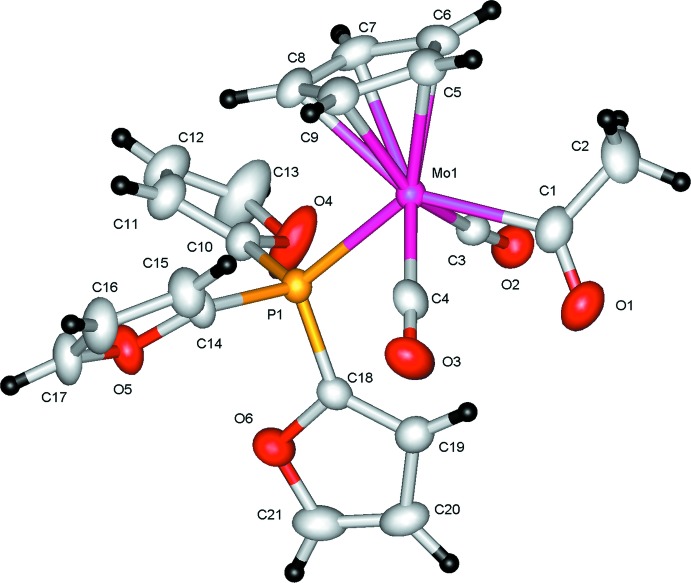
Molecular structure of I (50% probability ellipsoids for non-H atoms). H atoms are presented as small spheres of arbitrary radius.

**Fig. 2.**
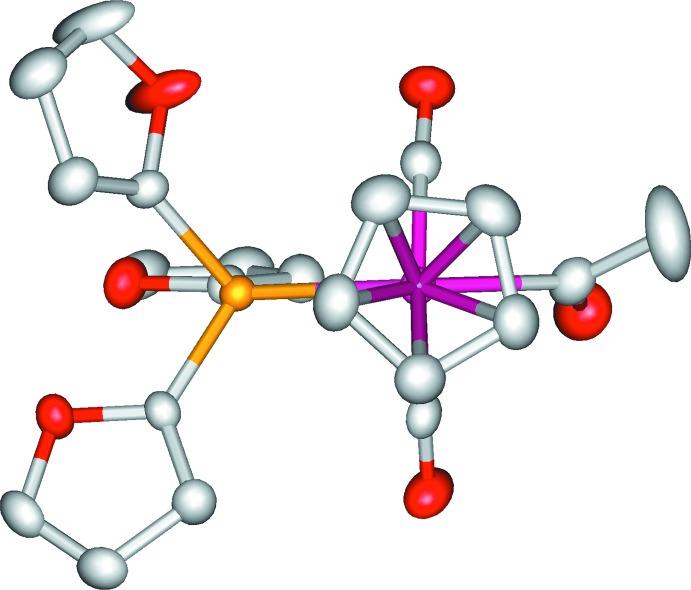
View of I perpendicular to Cp least-squares plane showing trans CO orientation.

**Fig. 3.**
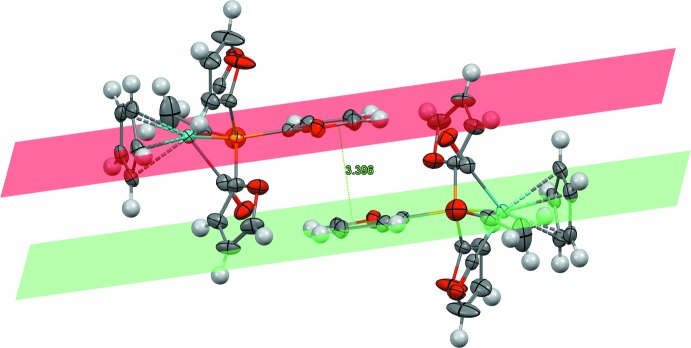
The centrosymmetrical dimers of I.

**Fig. 4.**
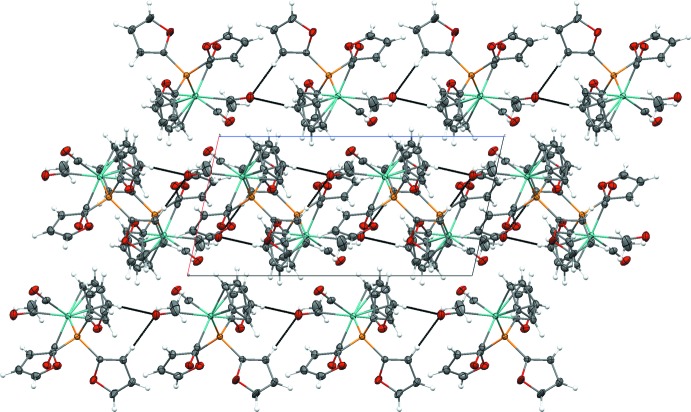
Crystal packing of I along the b axis showing the layers parallel to (100). Dashed lines indicate the intermolecular C—H···O hydrogen bonds.

## supplementary crystallographic information

### Comment

Synthesis of the title complex, [Mo(C_5_H_5_)(C_2_H_3_O)(CO)_2_(C_12_H_12_O_3_P)] (I), has not previously been reported, though several analogues containing various phosphine ligands have been reported and their reactivity studied (Adams *et al.*, 1997; Barnett *et al.*, 1972). The most closely related complexes, for which structural information is available, contain a triphenylphosphine or methyldiphenylphosphine ligand (Churchill & Fennessey, 1968; Whited *et al.*, 2012).

The molecular structure of I consists of a Mo(II) atom coordinated to a cyclopentadienyl ring in an *η*^5^ fashion, two CO ligands, one tris(furan-2-yl)phosphane ligand, and one acetyl ligand (Fig. 1, Table 1). The orientation of the CO ligands can be described as *trans* (Fig. 2). The Mo—Cp centroid distance is 2.029 (2) Å.

In the crystal, the molecules of I form centrosymmetrical dimers *via* the π—π interactions between furyl rings (the centroid-to-centroid distance is 3.396 (4) Å, Fig. 3).

There are several particularly short intermolecular distances involving H atoms. One short contact (2.38 Å) is present between O1 of the acetyl carbonyl on one Mo complex and H8 of a Cp ring on another (Table 2). Another short contact (2.40 Å) involves O1 of the acetyl group of one Mo complex and H11 of a furyl group on another (Table 2). These contacts between the acetyl carbonyl and hydrogen atoms may contribute to the unusual geometry adopted by the acetyl ligand, where the carbonyl points down, away from the Cp ring. In related structures reported by this laboratory (Whited *et al.*, 2012) and others (Churchill & Fennessey, 1968; Michelini-Rodriguez *et al.*, 1993; Adams *et al.*, 1997, 2000), the carbonyl points up toward the Cp ring. These hydrogen-bonding interactions lead to the formation of layers parallel to (100), as shown in Fig. 4.

### Experimental

CpMo(CO)_3_(CH_3_). This compound was prepared by a modification of the method used by Gladysz *et al.* (1979), as previously reported by Whited *et al.* (2012).

CpMo(CO)_2_(P(2-Fur)_3_)(COCH_3_) (I). In an inert-atmosphere glove box, CpMo(CO)_3_(CH_3_) (30.6 mg, 0.118 mmol) was dissolved in 2 ml acetonitrile. In a separate vial, tris(furan-2-yl)phosphane (42.0 mg, 0.181 mmol) was dissolved in 2 ml acetonitrile. The vials were combined and the resulting solution was stirred for 1 week. Solvent was removed *in vacuo*, leaving an orange oil that was triturated with pentane (5 mL) and isolated by filtration to afford the desired product in pure form as a yellow powder (21.6 mg, 37.2%), as confirmed by IR and NMR (^1^H, ^13^C, and ^31^P) spectral analyses. Crystalline material was obtained as yellow-orange prisms by vapor diffusion of pentane into a concentrated solution of I in diethyl ether at 233 K.

### Refinement

H-atoms were treated in calculated positions and refined in the riding model approximation with distances of C—H = 0.95, 1.00 and 0.98 Å for the furanyl, cyclopentadienyl and methyl groups, respectively, and with *U_iso_*(H) = *k*×*U_eq_*(C), *k* = 1.2 for furanyl and cyclopentadienyl groups and 1.5 for methyl groups. Methyl group H atoms were allowed to rotate in order to find the best rotameric conformation. The maximum and minimum electron densities in the final difference Fourier map are located 0.98 and 0.77 Å, respectively, from atom Mo1.

Eight low-angle reflections were rejected from the high-quality data set due to the arrangement of the instrument with a conservatively sized beam stop and a fixed-position detector. The large number of reflections in this data set (and the Fourier-transform relationship of intensities to atoms) ensures that no particular bias was thereby introduced into this routine structure determination.

### Crystal data

**Table:**

[Mo(C_5_H_5_)(C_2_H_3_O)(C_12_H_9_O_3_P)(CO)_2_]	*F*(000) = 992.00
*M**_r_* = 492.28	*D*_x_ = 1.644 Mg m^−^^3^
Monoclinic, *P*2_1_/*c*	Mo *K*α radiation, λ = 0.71075 Å
Hall symbol: -P 2ybc	Cell parameters from 13392 reflections
*a* = 8.050 (2) Å	θ = 3.2–27.6°
*b* = 15.762 (4) Å	µ = 0.78 mm^−^^1^
*c* = 16.073 (4) Å	*T* = 173 K
β = 102.852 (8)°	Prism, orange
*V* = 1988.4 (9) Å^3^	0.24 × 0.17 × 0.15 mm
*Z* = 4	

### Data collection

**Table:**

Rigaku XtaLAB mini diffractometer	3799 reflections with *F*^2^ > 2σ(*F*^2^)
Detector resolution: 6.827 pixels mm^-1^	*R*_int_ = 0.044
ω scans	θ_max_ = 27.5°
Absorption correction: multi-scan (*REQAB*; Rigaku, 1998)	*h* = −10→9
*T*_min_ = 0.709, *T*_max_ = 0.890	*k* = −20→20
16222 measured reflections	*l* = −20→20
4539 independent reflections	

### Refinement

**Table:**

Refinement on *F*^2^	Secondary atom site location: difference Fourier map
*R*[*F*^2^ > 2σ(*F*^2^)] = 0.035	Hydrogen site location: inferred from neighbouring sites
*wR*(*F*^2^) = 0.075	H-atom parameters constrained
*S* = 1.06	*w* = 1/[σ^2^(*F*_o_^2^) + (0.025*P*)^2^ + 1.9823*P*] where *P* = (*F*_o_^2^ + 2*F*_c_^2^)/3
4539 reflections	(Δ/σ)_max_ < 0.001
263 parameters	Δρ_max_ = 0.55 e Å^−^^3^
0 restraints	Δρ_min_ = −0.52 e Å^−^^3^
Primary atom site location: structure-invariant direct methods	

### Special details

**Table:**

Refinement. Refinement was performed using all reflections. The weighted *R*-factor (*wR*) and goodness of fit (*S*) are based on *F*^2^. *R*-factor (gt) are based on *F*. The threshold expression of *F*^2^ > 2.0 σ(*F*^2^) is used only for calculating *R*-factor (gt).

### Fractional atomic coordinates and isotropic or equivalent isotropic displacement parameters (Å^2^)

**Table:**

	*x*	*y*	*z*	*U*_iso_*/*U*_eq_	
Mo1	0.28931 (3)	0.203772 (14)	0.114671 (14)	0.01996 (7)	
P1	0.43789 (8)	0.33427 (4)	0.16296 (4)	0.02053 (15)	
O1	0.2805 (4)	0.17390 (16)	−0.07804 (14)	0.0472 (6)	
O2	0.6441 (3)	0.15779 (14)	0.07963 (13)	0.0357 (5)	
O3	0.1064 (3)	0.33025 (14)	−0.02625 (14)	0.0419 (6)	
O4	0.7480 (3)	0.2905 (2)	0.25444 (14)	0.0615 (9)	
O5	0.3794 (3)	0.49098 (13)	0.22768 (13)	0.0345 (5)	
O6	0.6466 (3)	0.45252 (13)	0.11535 (13)	0.0334 (5)	
C1	0.2624 (4)	0.1388 (2)	−0.01250 (19)	0.0338 (7)	
C2	0.2310 (7)	0.0460 (3)	−0.0174 (3)	0.0706 (14)	
C3	0.5144 (4)	0.17806 (17)	0.09207 (16)	0.0243 (6)	
C4	0.1775 (4)	0.28439 (17)	0.02493 (18)	0.0282 (7)	
C5	0.0773 (4)	0.11426 (19)	0.14393 (19)	0.0331 (7)	
C6	0.2380 (4)	0.08037 (19)	0.18492 (19)	0.0354 (7)	
C7	0.3150 (4)	0.1382 (2)	0.24888 (18)	0.0356 (7)	
C8	0.2035 (4)	0.2073 (2)	0.24670 (18)	0.0333 (7)	
C9	0.0548 (4)	0.19244 (19)	0.18202 (19)	0.0325 (7)	
C10	0.6004 (4)	0.32891 (18)	0.26045 (17)	0.0247 (6)	
C11	0.6050 (4)	0.3458 (2)	0.34274 (18)	0.0342 (7)	
C12	0.7657 (5)	0.3184 (3)	0.39118 (19)	0.0423 (9)	
C13	0.8462 (5)	0.2861 (4)	0.3364 (3)	0.0703 (15)	
C14	0.3049 (4)	0.41841 (17)	0.18893 (17)	0.0254 (6)	
C15	0.1340 (4)	0.4247 (2)	0.17555 (19)	0.0354 (7)	
C16	0.0987 (5)	0.5054 (2)	0.2072 (2)	0.0406 (8)	
C17	0.2480 (5)	0.5421 (2)	0.2373 (2)	0.0393 (8)	
C18	0.5519 (4)	0.38037 (17)	0.08986 (16)	0.0237 (6)	
C19	0.5672 (4)	0.35793 (19)	0.01093 (18)	0.0315 (7)	
C20	0.6773 (4)	0.4186 (2)	−0.0150 (2)	0.0374 (8)	
C21	0.7201 (4)	0.4734 (2)	0.0491 (2)	0.0392 (8)	
H2A	0.1192	0.0339	−0.0055	0.0847*	
H2B	0.3196	0.0170	0.0247	0.0847*	
H2C	0.2336	0.0256	−0.0747	0.0847*	
H5	−0.0110	0.0843	0.1003	0.0397*	
H6	0.2812	0.0224	0.1758	0.0425*	
H7	0.4236	0.1288	0.2922	0.0427*	
H8	0.2210	0.2559	0.2877	0.0399*	
H9	−0.0506	0.2280	0.1698	0.0390*	
H11	0.5168	0.3715	0.3646	0.0410*	
H12	0.8064	0.3227	0.4512	0.0507*	
H13	0.9576	0.2626	0.3511	0.0844*	
H15	0.0529	0.3833	0.1500	0.0425*	
H16	−0.0105	0.5285	0.2068	0.0487*	
H17	0.2620	0.5969	0.2624	0.0471*	
H19	0.5147	0.3108	−0.0213	0.0378*	
H20	0.7128	0.4196	−0.0675	0.0449*	
H21	0.7925	0.5210	0.0490	0.0470*	

### Atomic displacement parameters (Å^2^)

**Table:**

	*U*^11^	*U*^22^	*U*^33^	*U*^12^	*U*^13^	*U*^23^
Mo1	0.01978 (12)	0.02049 (12)	0.01972 (11)	−0.00095 (9)	0.00462 (8)	−0.00145 (9)
P1	0.0182 (4)	0.0218 (4)	0.0205 (4)	0.0010 (3)	0.0019 (3)	−0.0024 (3)
O1	0.0551 (16)	0.0590 (16)	0.0275 (12)	0.0048 (13)	0.0091 (11)	−0.0021 (11)
O2	0.0297 (12)	0.0416 (13)	0.0372 (12)	0.0077 (10)	0.0102 (10)	0.0013 (10)
O3	0.0439 (14)	0.0342 (12)	0.0386 (13)	−0.0032 (11)	−0.0101 (11)	0.0062 (10)
O4	0.0363 (14)	0.120 (3)	0.0239 (11)	0.0383 (15)	−0.0028 (10)	−0.0109 (13)
O5	0.0311 (11)	0.0289 (11)	0.0408 (12)	0.0030 (9)	0.0017 (10)	−0.0134 (9)
O6	0.0317 (12)	0.0309 (11)	0.0359 (12)	−0.0088 (9)	0.0038 (9)	−0.0006 (9)
C1	0.0271 (16)	0.0452 (19)	0.0288 (16)	−0.0002 (14)	0.0053 (13)	−0.0068 (14)
C2	0.108 (4)	0.050 (3)	0.065 (3)	−0.035 (3)	0.044 (3)	−0.029 (2)
C3	0.0289 (15)	0.0229 (14)	0.0211 (13)	−0.0022 (12)	0.0057 (11)	0.0016 (11)
C4	0.0254 (15)	0.0255 (15)	0.0310 (15)	−0.0065 (12)	0.0003 (12)	−0.0062 (12)
C5	0.0334 (17)	0.0321 (16)	0.0370 (16)	−0.0103 (13)	0.0148 (13)	−0.0029 (13)
C6	0.0445 (19)	0.0270 (16)	0.0406 (17)	0.0012 (14)	0.0218 (15)	0.0086 (13)
C7	0.0347 (17)	0.048 (2)	0.0257 (15)	0.0015 (15)	0.0112 (13)	0.0113 (14)
C8	0.0359 (17)	0.0427 (18)	0.0249 (14)	−0.0079 (15)	0.0147 (13)	−0.0057 (13)
C9	0.0267 (15)	0.0382 (17)	0.0363 (16)	−0.0044 (13)	0.0150 (13)	0.0002 (13)
C10	0.0189 (13)	0.0305 (15)	0.0243 (14)	0.0023 (11)	0.0039 (11)	−0.0020 (11)
C11	0.0296 (16)	0.0474 (19)	0.0258 (15)	0.0029 (14)	0.0068 (13)	−0.0042 (13)
C12	0.0366 (18)	0.064 (3)	0.0228 (15)	0.0043 (16)	−0.0011 (13)	0.0006 (15)
C13	0.039 (2)	0.138 (5)	0.0272 (17)	0.041 (3)	−0.0083 (16)	−0.006 (3)
C14	0.0267 (15)	0.0216 (14)	0.0265 (14)	0.0027 (12)	0.0028 (11)	−0.0060 (11)
C15	0.0245 (15)	0.0375 (18)	0.0432 (18)	0.0015 (14)	0.0052 (13)	−0.0118 (14)
C16	0.0329 (17)	0.047 (2)	0.0402 (18)	0.0157 (15)	0.0039 (14)	−0.0132 (15)
C17	0.047 (2)	0.0330 (17)	0.0368 (17)	0.0138 (15)	0.0060 (15)	−0.0141 (14)
C18	0.0226 (14)	0.0218 (13)	0.0249 (13)	−0.0017 (11)	0.0012 (11)	0.0012 (11)
C19	0.0364 (17)	0.0312 (16)	0.0269 (15)	0.0001 (14)	0.0069 (13)	0.0021 (12)
C20	0.0359 (17)	0.0452 (19)	0.0335 (16)	0.0007 (15)	0.0124 (14)	0.0131 (15)
C21	0.0298 (17)	0.0414 (19)	0.0445 (19)	−0.0075 (15)	0.0045 (14)	0.0147 (15)

### Geometric parameters (Å, º)

**Table:**

Mo1—P1	2.4189 (8)	C10—C11	1.342 (4)
Mo1—C1	2.253 (4)	C11—C12	1.421 (5)
Mo1—C3	1.968 (3)	C12—C13	1.307 (6)
Mo1—C4	1.982 (3)	C14—C15	1.348 (5)
Mo1—C5	2.341 (4)	C15—C16	1.422 (5)
Mo1—C6	2.332 (4)	C16—C17	1.325 (5)
Mo1—C7	2.359 (3)	C18—C19	1.349 (4)
Mo1—C8	2.374 (4)	C19—C20	1.427 (5)
Mo1—C9	2.382 (4)	C20—C21	1.330 (5)
P1—C10	1.806 (3)	C2—H2A	0.980
P1—C14	1.810 (3)	C2—H2B	0.980
P1—C18	1.797 (3)	C2—H2C	0.980
O1—C1	1.227 (4)	C5—H5	1.000
O2—C3	1.150 (4)	C6—H6	1.000
O3—C4	1.148 (4)	C7—H7	1.000
O4—C10	1.356 (4)	C8—H8	1.000
O4—C13	1.379 (4)	C9—H9	1.000
O5—C14	1.375 (4)	C11—H11	0.950
O5—C17	1.365 (4)	C12—H12	0.950
O6—C18	1.380 (4)	C13—H13	0.950
O6—C21	1.368 (5)	C15—H15	0.950
C1—C2	1.484 (6)	C16—H16	0.950
C5—C6	1.419 (5)	C17—H17	0.950
C5—C9	1.406 (5)	C19—H19	0.950
C6—C7	1.410 (5)	C20—H20	0.950
C7—C8	1.406 (5)	C21—H21	0.950
C8—C9	1.420 (4)		
			
P1···O3	3.573 (3)	C2···H5^xiii^	2.8413
O1···O2	3.428 (3)	C2···H11^i^	3.5382
O1···O3	3.041 (4)	C3···H9^iv^	3.5348
O1···C3	2.956 (4)	C3···H17^v^	2.9114
O1···C4	2.659 (4)	C4···H12^ii^	3.4063
O2···O4	3.453 (4)	C4···H13^ii^	3.0478
O2···C1	3.114 (4)	C4···H21^vi^	3.3179
O2···C18	3.597 (4)	C5···H2A^xiii^	3.3681
O2···C19	3.354 (4)	C5···H2C^xiii^	3.3355
O3···C1	3.258 (4)	C5···H12^ii^	3.5118
O3···C15	3.531 (4)	C5···H16^ix^	2.9065
O4···O6	3.373 (4)	C5···H17^ix^	3.4107
O4···C3	3.362 (4)	C6···H16^ix^	2.9100
O4···C18	3.105 (4)	C7···H16^ix^	3.2069
O5···O6	3.160 (4)	C7···H17^v^	3.5114
O5···C10	3.091 (4)	C8···H13^viii^	2.9953
O5···C11	3.234 (4)	C8···H16^ix^	3.3813
O5···C18	3.353 (4)	C9···H13^viii^	3.1884
O6···C10	3.124 (4)	C9···H16^ix^	3.2067
O6···C14	3.271 (4)	C9···H17^ix^	3.2533
C2···C3	3.294 (5)	C10···H6^vii^	3.2903
C2···C5	3.293 (6)	C11···H2B^vii^	3.4072
C2···C6	3.285 (6)	C11···H6^vii^	2.9657
C3···C10	3.553 (4)	C11···H19^x^	3.4790
C3···C18	3.204 (4)	C12···H2B^vii^	3.5370
C3···C19	3.188 (5)	C12···H6^vii^	3.3854
C4···C14	3.356 (4)	C12···H19^x^	3.3869
C4···C15	3.355 (5)	C13···H8^iv^	3.3152
C4···C18	3.326 (4)	C13···H9^iv^	3.1123
C4···C19	3.398 (5)	C13···H17^v^	3.3987
C8···C14	3.597 (5)	C14···H20^vi^	3.1976
C11···C14	3.255 (4)	C15···H20^vi^	3.3925
O1···C8^i^	3.324 (4)	C15···H21^viii^	3.3930
O1···C11^i^	3.166 (5)	C16···H2C^x^	3.4656
O1···C13^ii^	3.518 (5)	C16···H5^xii^	3.5488
O2···C2^iii^	3.574 (5)	C16···H20^vi^	3.2027
O2···C5^iv^	3.479 (4)	C16···H21^viii^	3.1327
O2···C9^iv^	3.394 (4)	C17···H2C^x^	3.2303
O2···C12^i^	3.405 (5)	C17···H7^vii^	3.1047
O2···C17^v^	3.406 (4)	C17···H20^vi^	2.8803
O3···C13^ii^	3.250 (5)	C18···H21^vi^	3.5115
O3···C21^vi^	3.449 (4)	C19···H7^i^	3.4546
O4···C9^iv^	3.335 (5)	C19···H21^vi^	3.4225
O5···C6^vii^	3.395 (4)	C20···H7^i^	3.3946
O5···C7^vii^	3.342 (4)	C20···H15^iv^	3.5936
O6···C20^vi^	3.414 (4)	C21···H15^iv^	3.1450
C2···O2^iii^	3.574 (5)	C21···H16^iv^	3.0711
C5···O2^viii^	3.479 (4)	H2A···C2^xiii^	3.1828
C5···C16^ix^	3.491 (5)	H2A···C5^xiii^	3.3681
C6···O5^v^	3.395 (4)	H2A···H2A^xiii^	2.2378
C7···O5^v^	3.342 (4)	H2A···H2B^xiii^	3.5677
C8···O1^x^	3.324 (4)	H2A···H2C^xiii^	3.4984
C9···O2^viii^	3.394 (4)	H2A···H5^xiii^	2.4410
C9···O4^viii^	3.335 (5)	H2A···H12^v^	3.4606
C11···O1^x^	3.166 (5)	H2A···H12^ii^	3.3393
C12···O2^x^	3.405 (5)	H2B···O2^iii^	3.2721
C13···O1^xi^	3.518 (5)	H2B···C11^v^	3.4072
C13···O3^xi^	3.250 (5)	H2B···C12^v^	3.5370
C15···C21^viii^	3.579 (4)	H2B···H2A^xiii^	3.5677
C16···C5^xii^	3.491 (5)	H2B···H2B^iii^	3.2227
C16···C21^viii^	3.543 (5)	H2B···H2C^iii^	3.5712
C17···O2^vii^	3.406 (4)	H2B···H5^xiii^	3.2452
C18···C21^vi^	3.595 (4)	H2B···H11^v^	3.0188
C19···C21^vi^	3.515 (5)	H2B···H12^v^	3.2762
C20···O6^vi^	3.414 (4)	H2C···O2^iii^	3.0612
C20···C21^vi^	3.557 (5)	H2C···C5^xiii^	3.3355
C21···O3^vi^	3.449 (4)	H2C···C16^i^	3.4656
C21···C15^iv^	3.579 (4)	H2C···C17^i^	3.2303
C21···C16^iv^	3.543 (5)	H2C···H2A^xiii^	3.4984
C21···C18^vi^	3.595 (4)	H2C···H2B^iii^	3.5712
C21···C19^vi^	3.515 (5)	H2C···H5^xiii^	2.4602
C21···C20^vi^	3.557 (5)	H2C···H11^i^	3.1246
Mo1···H2A	3.4052	H2C···H17^i^	3.3020
Mo1···H2B	3.3128	H5···O2^viii^	2.9591
Mo1···H15	3.5256	H5···C2^xiii^	2.8413
Mo1···H19	3.5603	H5···C16^ix^	3.5488
P1···H8	3.1866	H5···H2A^xiii^	2.4410
P1···H11	3.2156	H5···H2B^xiii^	3.2452
P1···H15	3.1555	H5···H2C^xiii^	2.4602
P1···H19	3.1787	H5···H12^ii^	2.9127
O1···H2A	2.9305	H5···H16^ix^	3.1909
O1···H2B	2.9517	H5···H17^ix^	3.3139
O1···H2C	2.3698	H6···O5^v^	2.8696
O1···H19	2.8766	H6···O6^v^	3.4567
O2···H2B	3.3917	H6···C10^v^	3.2903
O2···H19	2.9626	H6···C11^v^	2.9657
O3···H15	3.0736	H6···C12^v^	3.3854
O3···H19	3.2846	H6···H11^v^	3.0322
O4···H11	3.1144	H6···H16^ix^	3.1850
O4···H12	3.1337	H7···O5^v^	2.7503
O5···H11	2.9209	H7···O6^v^	3.2600
O5···H15	3.1445	H7···C17^v^	3.1047
O5···H16	3.1378	H7···C19^x^	3.4546
O6···H19	3.1475	H7···C20^x^	3.3946
O6···H20	3.1439	H7···H17^v^	2.9010
C1···H5	3.2622	H7···H19^x^	3.0750
C1···H6	3.5136	H7···H20^x^	2.9593
C1···H19	3.4091	H8···O1^x^	2.3768
C2···H5	3.0620	H8···O3^x^	3.5861
C2···H6	3.0642	H8···C1^x^	3.5639
C3···H2B	3.0535	H8···C13^viii^	3.3152
C3···H6	3.5333	H8···H13^viii^	2.5503
C3···H7	3.5392	H8···H19^x^	3.5900
C3···H19	2.7741	H9···O2^viii^	2.7898
C4···H9	3.3890	H9···O4^viii^	2.5358
C4···H15	2.8933	H9···C3^viii^	3.5348
C4···H19	2.9982	H9···C13^viii^	3.1123
C5···H2A	2.8007	H9···H12^ii^	3.5362
C5···H2B	3.3893	H9···H13^viii^	2.9508
C5···H7	3.2477	H9···H17^ix^	3.0301
C5···H8	3.2364	H11···O1^x^	2.4000
C6···H2A	3.0845	H11···O2^x^	3.4100
C6···H2B	2.9686	H11···C1^x^	3.1494
C6···H8	3.2410	H11···C2^x^	3.5382
C6···H9	3.2593	H11···H2B^vii^	3.0188
C7···H5	3.2448	H11···H2C^x^	3.1246
C7···H9	3.2588	H11···H6^vii^	3.0322
C8···H5	3.2368	H11···H19^x^	3.4104
C8···H6	3.2403	H12···O2^x^	2.6951
C8···H15	3.2779	H12···O3^xi^	3.3742
C9···H6	3.2549	H12···C4^xi^	3.4063
C9···H7	3.2582	H12···C5^xi^	3.5118
C9···H15	3.0516	H12···H2A^vii^	3.4606
C10···H7	3.5430	H12···H2A^xi^	3.3393
C10···H8	3.3826	H12···H2B^vii^	3.2762
C10···H12	3.1464	H12···H5^xi^	2.9127
C10···H13	3.1018	H12···H9^xi^	3.5362
C11···H8	3.3365	H12···H19^x^	3.2551
C11···H13	3.1033	H13···O1^xi^	2.7818
C13···H11	3.0967	H13···O3^xi^	2.5324
C14···H8	3.1651	H13···C1^xi^	3.2942
C14···H11	3.0477	H13···C4^xi^	3.0478
C14···H16	3.1411	H13···C8^iv^	2.9953
C14···H17	3.1002	H13···C9^iv^	3.1884
C15···H8	3.2035	H13···H8^iv^	2.5503
C15···H9	3.4305	H13···H9^iv^	2.9508
C15···H17	3.1200	H13···H17^v^	3.4406
C17···H15	3.1177	H15···O4^viii^	3.5809
C18···H20	3.1487	H15···O6^viii^	3.3749
C18···H21	3.1083	H15···C20^viii^	3.5936
C19···H21	3.1265	H15···C21^viii^	3.1450
C21···H19	3.1235	H15···H21^viii^	3.1980
H2A···H5	2.3253	H16···O6^viii^	3.0672
H2A···H6	2.9215	H16···C5^xii^	2.9065
H2B···H5	3.3341	H16···C6^xii^	2.9100
H2B···H6	2.5171	H16···C7^xii^	3.2069
H5···H6	2.5858	H16···C8^xii^	3.3813
H5···H9	2.5780	H16···C9^xii^	3.2067
H6···H7	2.5833	H16···C21^viii^	3.0711
H7···H8	2.5739	H16···H5^xii^	3.1909
H8···H9	2.5930	H16···H6^xii^	3.1850
H8···H11	3.0352	H16···H21^viii^	2.6784
H8···H15	3.0715	H17···O2^vii^	2.6622
H9···H15	2.6274	H17···O4^vii^	3.0622
H11···H12	2.5539	H17···C3^vii^	2.9114
H12···H13	2.4165	H17···C5^xii^	3.4107
H15···H16	2.5576	H17···C7^vii^	3.5114
H16···H17	2.4292	H17···C9^xii^	3.2533
H19···H20	2.5627	H17···C13^vii^	3.3987
H20···H21	2.4355	H17···H2C^x^	3.3020
O1···H8^i^	2.3768	H17···H5^xii^	3.3139
O1···H11^i^	2.4000	H17···H7^vii^	2.9010
O1···H13^ii^	2.7818	H17···H9^xii^	3.0301
O2···H2B^iii^	3.2721	H17···H13^vii^	3.4406
O2···H2C^iii^	3.0612	H17···H20^vi^	3.1945
O2···H5^iv^	2.9591	H19···C11^i^	3.4790
O2···H9^iv^	2.7898	H19···C12^i^	3.3869
O2···H11^i^	3.4100	H19···H7^i^	3.0750
O2···H12^i^	2.6951	H19···H8^i^	3.5900
O2···H17^v^	2.6622	H19···H11^i^	3.4104
O3···H8^i^	3.5861	H19···H12^i^	3.2551
O3···H12^ii^	3.3742	H19···H21^vi^	3.5859
O3···H13^ii^	2.5324	H20···O3^iv^	3.3953
O3···H20^viii^	3.3953	H20···O5^vi^	2.8838
O3···H21^vi^	2.5352	H20···O6^vi^	3.4695
O4···H9^iv^	2.5358	H20···C14^vi^	3.1976
O4···H15^iv^	3.5809	H20···C15^vi^	3.3925
O4···H17^v^	3.0622	H20···C16^vi^	3.2027
O5···H6^vii^	2.8696	H20···C17^vi^	2.8803
O5···H7^vii^	2.7503	H20···H7^i^	2.9593
O5···H20^vi^	2.8838	H20···H17^vi^	3.1945
O6···H6^vii^	3.4567	H21···O3^vi^	2.5352
O6···H7^vii^	3.2600	H21···C4^vi^	3.3179
O6···H15^iv^	3.3749	H21···C15^iv^	3.3930
O6···H16^iv^	3.0672	H21···C16^iv^	3.1327
O6···H20^vi^	3.4695	H21···C18^vi^	3.5115
C1···H8^i^	3.5639	H21···C19^vi^	3.4225
C1···H11^i^	3.1494	H21···H15^iv^	3.1980
C1···H13^ii^	3.2942	H21···H16^iv^	2.6784
C2···H2A^xiii^	3.1828	H21···H19^vi^	3.5859
			
P1—Mo1—C1	128.21 (9)	C7—C8—C9	108.7 (3)
P1—Mo1—C3	79.89 (8)	Mo1—C9—C5	71.1 (2)
P1—Mo1—C4	78.48 (8)	Mo1—C9—C8	72.30 (19)
P1—Mo1—C5	141.13 (8)	C5—C9—C8	107.1 (3)
P1—Mo1—C6	133.02 (8)	P1—C10—O4	115.9 (2)
P1—Mo1—C7	98.20 (8)	P1—C10—C11	134.6 (3)
P1—Mo1—C8	85.10 (8)	O4—C10—C11	109.1 (3)
P1—Mo1—C9	107.65 (8)	C10—C11—C12	107.5 (3)
C1—Mo1—C3	69.24 (11)	C11—C12—C13	106.1 (3)
C1—Mo1—C4	72.24 (12)	O4—C13—C12	111.0 (4)
C1—Mo1—C5	88.91 (12)	P1—C14—O5	119.6 (2)
C1—Mo1—C6	93.91 (12)	P1—C14—C15	130.7 (3)
C1—Mo1—C7	126.97 (12)	O5—C14—C15	109.7 (3)
C1—Mo1—C8	146.42 (12)	C14—C15—C16	106.7 (3)
C1—Mo1—C9	116.88 (11)	C15—C16—C17	106.4 (3)
C3—Mo1—C4	106.58 (12)	O5—C17—C16	111.4 (3)
C3—Mo1—C5	130.94 (11)	P1—C18—O6	117.9 (2)
C3—Mo1—C6	100.79 (12)	P1—C18—C19	132.5 (3)
C3—Mo1—C7	100.46 (11)	O6—C18—C19	109.6 (3)
C3—Mo1—C8	129.25 (10)	C18—C19—C20	106.9 (3)
C3—Mo1—C9	157.46 (11)	C19—C20—C21	106.3 (3)
C4—Mo1—C5	107.42 (11)	O6—C21—C20	111.3 (3)
C4—Mo1—C6	141.87 (11)	C1—C2—H2A	109.475
C4—Mo1—C7	151.60 (13)	C1—C2—H2B	109.476
C4—Mo1—C8	117.41 (12)	C1—C2—H2C	109.471
C4—Mo1—C9	95.82 (12)	H2A—C2—H2B	109.480
C5—Mo1—C6	35.35 (11)	H2A—C2—H2C	109.467
C5—Mo1—C7	58.13 (11)	H2B—C2—H2C	109.459
C5—Mo1—C8	57.63 (11)	Mo1—C5—H5	125.465
C5—Mo1—C9	34.61 (11)	C6—C5—H5	125.462
C6—Mo1—C7	34.99 (11)	C9—C5—H5	125.463
C6—Mo1—C8	57.89 (11)	Mo1—C6—H6	125.921
C6—Mo1—C9	58.25 (11)	C5—C6—H6	125.931
C7—Mo1—C8	34.57 (11)	C7—C6—H6	125.919
C7—Mo1—C9	57.92 (11)	Mo1—C7—H7	125.887
C8—Mo1—C9	34.73 (10)	C6—C7—H7	125.888
Mo1—P1—C10	116.72 (10)	C8—C7—H7	125.885
Mo1—P1—C14	114.92 (10)	Mo1—C8—H8	125.541
Mo1—P1—C18	114.96 (9)	C7—C8—H8	125.535
C10—P1—C14	100.70 (13)	C9—C8—H8	125.540
C10—P1—C18	102.09 (13)	Mo1—C9—H9	126.321
C14—P1—C18	105.58 (13)	C5—C9—H9	126.319
C10—O4—C13	106.3 (3)	C8—C9—H9	126.314
C14—O5—C17	105.8 (3)	C10—C11—H11	126.232
C18—O6—C21	105.9 (3)	C12—C11—H11	126.226
Mo1—C1—O1	124.6 (3)	C11—C12—H12	126.961
Mo1—C1—C2	118.5 (3)	C13—C12—H12	126.951
O1—C1—C2	116.8 (3)	O4—C13—H13	124.493
Mo1—C3—O2	175.7 (3)	C12—C13—H13	124.484
Mo1—C4—O3	177.1 (3)	C14—C15—H15	126.643
Mo1—C5—C6	71.96 (19)	C16—C15—H15	126.650
Mo1—C5—C9	74.30 (19)	C15—C16—H16	126.781
C6—C5—C9	108.7 (3)	C17—C16—H16	126.780
Mo1—C6—C5	72.69 (18)	O5—C17—H17	124.313
Mo1—C6—C7	73.56 (19)	C16—C17—H17	124.310
C5—C6—C7	107.6 (3)	C18—C19—H19	126.546
Mo1—C7—C6	71.45 (18)	C20—C19—H19	126.540
Mo1—C7—C8	73.29 (18)	C19—C20—H20	126.865
C6—C7—C8	107.9 (3)	C21—C20—H20	126.858
Mo1—C8—C7	72.14 (19)	O6—C21—H21	124.340
Mo1—C8—C9	72.97 (19)	C20—C21—H21	124.322
			
P1—Mo1—C1—O1	−21.0 (3)	C8—Mo1—C5—C6	−78.82 (14)
P1—Mo1—C1—C2	155.15 (13)	C8—Mo1—C5—C9	37.38 (11)
C1—Mo1—P1—C10	−123.99 (11)	C5—Mo1—C9—C5	0.00 (12)
C1—Mo1—P1—C14	118.42 (11)	C5—Mo1—C9—C8	115.8 (2)
C1—Mo1—P1—C18	−4.46 (11)	C9—Mo1—C5—C6	−116.2 (2)
C3—Mo1—P1—C10	−70.77 (9)	C9—Mo1—C5—C9	0.00 (12)
C3—Mo1—P1—C14	171.65 (8)	C6—Mo1—C7—C6	0.00 (15)
C3—Mo1—P1—C18	48.76 (8)	C6—Mo1—C7—C8	−116.1 (3)
C4—Mo1—P1—C10	179.83 (10)	C7—Mo1—C6—C5	115.0 (3)
C4—Mo1—P1—C14	62.25 (10)	C7—Mo1—C6—C7	0.00 (15)
C4—Mo1—P1—C18	−60.64 (10)	C6—Mo1—C8—C7	37.45 (12)
P1—Mo1—C5—C6	−97.48 (14)	C6—Mo1—C8—C9	−79.32 (14)
P1—Mo1—C5—C9	18.72 (19)	C8—Mo1—C6—C5	77.99 (15)
C5—Mo1—P1—C10	76.30 (12)	C8—Mo1—C6—C7	−36.99 (12)
C5—Mo1—P1—C14	−41.29 (12)	C6—Mo1—C9—C5	−37.63 (11)
C5—Mo1—P1—C18	−164.17 (11)	C6—Mo1—C9—C8	78.21 (13)
P1—Mo1—C6—C5	121.66 (9)	C9—Mo1—C6—C5	36.82 (12)
P1—Mo1—C6—C7	6.7 (2)	C9—Mo1—C6—C7	−78.17 (15)
C6—Mo1—P1—C10	24.61 (13)	C7—Mo1—C8—C7	0.00 (14)
C6—Mo1—P1—C14	−92.97 (12)	C7—Mo1—C8—C9	−116.8 (3)
C6—Mo1—P1—C18	144.14 (12)	C8—Mo1—C7—C6	116.1 (3)
P1—Mo1—C7—C6	−175.07 (11)	C8—Mo1—C7—C8	0.00 (13)
P1—Mo1—C7—C8	68.84 (12)	C7—Mo1—C9—C5	−79.11 (14)
C7—Mo1—P1—C10	28.47 (10)	C7—Mo1—C9—C8	36.73 (12)
C7—Mo1—P1—C14	−89.11 (9)	C9—Mo1—C7—C6	79.19 (15)
C7—Mo1—P1—C18	148.00 (9)	C9—Mo1—C7—C8	−36.89 (12)
P1—Mo1—C8—C7	−112.11 (11)	C8—Mo1—C9—C5	−115.8 (3)
P1—Mo1—C8—C9	131.13 (12)	C8—Mo1—C9—C8	−0.00 (14)
C8—Mo1—P1—C10	60.56 (8)	C9—Mo1—C8—C7	116.8 (3)
C8—Mo1—P1—C14	−57.03 (8)	C9—Mo1—C8—C9	−0.00 (13)
C8—Mo1—P1—C18	−179.91 (8)	Mo1—P1—C10—O4	74.1 (2)
P1—Mo1—C9—C5	−167.80 (8)	Mo1—P1—C10—C11	−96.6 (3)
P1—Mo1—C9—C8	−51.96 (12)	Mo1—P1—C14—O5	171.31 (15)
C9—Mo1—P1—C10	87.32 (8)	Mo1—P1—C14—C15	−9.8 (3)
C9—Mo1—P1—C14	−30.26 (8)	Mo1—P1—C18—O6	−176.72 (13)
C9—Mo1—P1—C18	−153.15 (8)	Mo1—P1—C18—C19	2.5 (3)
C3—Mo1—C1—O1	−78.4 (3)	C10—P1—C14—O5	45.0 (3)
C3—Mo1—C1—C2	97.7 (2)	C10—P1—C14—C15	−136.2 (3)
C4—Mo1—C1—O1	37.8 (3)	C14—P1—C10—O4	−160.81 (19)
C4—Mo1—C1—C2	−146.1 (3)	C14—P1—C10—C11	28.5 (3)
C1—Mo1—C5—C6	98.33 (14)	C10—P1—C18—O6	−49.4 (2)
C1—Mo1—C5—C9	−145.47 (13)	C10—P1—C18—C19	129.9 (3)
C5—Mo1—C1—O1	146.5 (3)	C18—P1—C10—O4	−52.1 (3)
C5—Mo1—C1—C2	−37.42 (19)	C18—P1—C10—C11	137.2 (3)
C1—Mo1—C6—C5	−82.56 (14)	C14—P1—C18—O6	55.5 (2)
C1—Mo1—C6—C7	162.46 (14)	C14—P1—C18—C19	−125.2 (3)
C6—Mo1—C1—O1	−178.5 (3)	C18—P1—C14—O5	−60.9 (2)
C6—Mo1—C1—C2	−2.4 (2)	C18—P1—C14—C15	117.9 (3)
C1—Mo1—C7—C6	−22.1 (2)	C10—O4—C13—C12	0.9 (5)
C1—Mo1—C7—C8	−138.20 (13)	C13—O4—C10—P1	−174.4 (3)
C7—Mo1—C1—O1	−166.0 (2)	C13—O4—C10—C11	−1.4 (4)
C7—Mo1—C1—C2	10.1 (3)	C14—O5—C17—C16	0.1 (3)
C1—Mo1—C8—C7	74.4 (3)	C17—O5—C14—P1	178.9 (2)
C1—Mo1—C8—C9	−42.4 (3)	C17—O5—C14—C15	−0.2 (3)
C8—Mo1—C1—O1	150.83 (19)	C18—O6—C21—C20	−0.3 (3)
C8—Mo1—C1—C2	−33.1 (3)	C21—O6—C18—P1	179.48 (19)
C1—Mo1—C9—C5	39.45 (16)	C21—O6—C18—C19	0.1 (3)
C1—Mo1—C9—C8	155.29 (12)	Mo1—C5—C6—Mo1	0.0
C9—Mo1—C1—O1	125.3 (3)	Mo1—C5—C6—C7	65.80 (19)
C9—Mo1—C1—C2	−58.6 (2)	Mo1—C5—C9—Mo1	0.0
C3—Mo1—C5—C6	37.40 (19)	Mo1—C5—C9—C8	−63.79 (19)
C3—Mo1—C5—C9	153.60 (11)	C6—C5—C9—Mo1	64.2 (3)
C3—Mo1—C6—C5	−152.15 (13)	C6—C5—C9—C8	0.4 (4)
C3—Mo1—C6—C7	92.86 (14)	C9—C5—C6—Mo1	−65.8 (3)
C3—Mo1—C7—C6	−93.92 (14)	C9—C5—C6—C7	0.0 (4)
C3—Mo1—C7—C8	150.00 (13)	Mo1—C6—C7—Mo1	0.0
C3—Mo1—C8—C7	−39.42 (19)	Mo1—C6—C7—C8	64.70 (19)
C3—Mo1—C8—C9	−156.19 (12)	C5—C6—C7—Mo1	−65.2 (3)
C3—Mo1—C9—C5	−61.2 (3)	C5—C6—C7—C8	−0.5 (4)
C3—Mo1—C9—C8	54.6 (4)	Mo1—C7—C8—Mo1	0.0
C4—Mo1—C5—C6	169.32 (13)	Mo1—C7—C8—C9	64.30 (19)
C4—Mo1—C5—C9	−74.48 (14)	C6—C7—C8—Mo1	−63.5 (3)
C4—Mo1—C6—C5	−16.6 (3)	C6—C7—C8—C9	0.8 (4)
C4—Mo1—C6—C7	−131.62 (17)	Mo1—C8—C9—Mo1	0.0
C4—Mo1—C7—C6	104.0 (3)	Mo1—C8—C9—C5	62.99 (19)
C4—Mo1—C7—C8	−12.1 (3)	C7—C8—C9—Mo1	−63.8 (3)
C4—Mo1—C8—C7	173.56 (11)	C7—C8—C9—C5	−0.8 (4)
C4—Mo1—C8—C9	56.80 (17)	P1—C10—C11—C12	172.5 (3)
C4—Mo1—C9—C5	112.47 (13)	O4—C10—C11—C12	1.4 (4)
C4—Mo1—C9—C8	−131.70 (13)	C10—C11—C12—C13	−0.8 (4)
C5—Mo1—C6—C5	0.00 (14)	C11—C12—C13—O4	−0.0 (5)
C5—Mo1—C6—C7	−115.0 (3)	P1—C14—C15—C16	−178.7 (2)
C6—Mo1—C5—C6	0.00 (14)	O5—C14—C15—C16	0.2 (3)
C6—Mo1—C5—C9	116.2 (3)	C14—C15—C16—C17	−0.1 (4)
C5—Mo1—C7—C6	38.14 (13)	C15—C16—C17—O5	−0.0 (4)
C5—Mo1—C7—C8	−77.95 (15)	P1—C18—C19—C20	−179.13 (19)
C7—Mo1—C5—C6	−37.74 (12)	O6—C18—C19—C20	0.1 (3)
C7—Mo1—C5—C9	78.46 (14)	C18—C19—C20—C21	−0.3 (4)
C5—Mo1—C8—C7	79.52 (14)	C19—C20—C21—O6	0.4 (4)
C5—Mo1—C8—C9	−37.25 (12)		

Symmetry codes: (i) *x*, −*y*+1/2, *z*−1/2; (ii) *x*−1, −*y*+1/2, *z*−1/2; (iii) −*x*+1, −*y*, −*z*; (iv) *x*+1, *y*, *z*; (v) −*x*+1, *y*−1/2, −*z*+1/2; (vi) −*x*+1, −*y*+1, −*z*; (vii) −*x*+1, *y*+1/2, −*z*+1/2; (viii) *x*−1, *y*, *z*; (ix) −*x*, *y*−1/2, −*z*+1/2; (x) *x*, −*y*+1/2, *z*+1/2; (xi) *x*+1, −*y*+1/2, *z*+1/2; (xii) −*x*, *y*+1/2, −*z*+1/2; (xiii) −*x*, −*y*, −*z*.

### Hydrogen-bond geometry (Å, º)

**Table:**

*D*—H···*A*	*D*—H	H···*A*	*D*···*A*	*D*—H···*A*
C8—H8···O1^x^	1.00	2.38	3.324 (4)	158
C11—H11···O1^x^	0.95	2.40	3.166 (5)	137

Symmetry code: (x) *x*, −*y*+1/2, *z*+1/2.
